# Interleukin-1α as a Potential Prognostic Biomarker in Pancreatic Cancer

**DOI:** 10.3390/biomedicines12061216

**Published:** 2024-05-30

**Authors:** Leonardo Gigante, Gwladys Gaudillière-Le Dain, Aurélie Bertaut, Caroline Truntzer, François Ghiringhelli

**Affiliations:** 1 Platform of Transfer in Biological Oncology, Georges François Leclerc Cancer Center-Unicancer, 1 Rue du Professeur Marion, 21000 Dijon, Francegghiringhelli@cgfl.fr (F.G.); 2 UFR of Health Sciences, University of Burgundy, 21000 Dijon, France; 3 Biostatistics and Methodology Unit, Georges-François Leclerc Cancer Center, 21000 Dijon, France; abertaut@cgfl.fr; 4 UMR INSERM 1231, 7 Boulevard Jeanne d’Arc, 21000 Dijon, France; 5 Genomic and Immunotherapy Medical Institute, Dijon University Hospital, 14 Rue Paul Gaffarel, 21000 Dijon, France; 6 Department of Medical Oncology, Georges-François Leclerc Cancer Center, 1 Rue du Professeur Marion, 21000 Dijon, France

**Keywords:** pancreatic cancer, inflammation, prognostic biomarker, interleukin 1

## Abstract

Purpose: We assessed the prognostic role of pro-inflammatory cytokines of the IL-1 superfamily in patients with pancreatic cancer. Methods: This retrospective study was performed using two independent cohorts of patients with pancreatic cancer: the International Cancer Genome Consortium (ICGC, N = 267) cohort and The Cancer Genome Atlas (TCGA, N = 178) cohort. Univariate Cox regressions were used to identify prognosis-related pro-inflammatory cytokines of the IL-1 superfamily. Cytokines associated with outcome were included in a multivariate Cox model with relevant clinicopathological variables to identify prognostic biomarkers. Results: IL-1α was the only pro-inflammatory cytokine of the IL-1 superfamily that was significantly associated with prognosis in both cohorts. In the training cohort (ICGC), the decile of patients with the lowest *IL1A* expression had better overall survival (HR = 1.99 [1.01–3.93], *p* = 0.05) and better relapse-free survival (HR = 1.85 [1.02–3.34], *p* = 0.04) than the group with the highest *IL1A* expression. The validation cohort (TCGA) confirmed these results: the decile with the lowest *IL1A* expression had better overall survival (HR = 3.00 [1.14–7.90], *p* = 0.03) and a lower risk of progression (HR = 3.11 [1.24–7.80], *p* = 0.01). Conclusions: *IL1A* is an independent prognostic marker and could be considered a potential therapeutic target in pancreatic cancer patients.

## 1. Introduction

The incidence of pancreatic cancer in France is growing (from 6000 cases/year in 2006 to more than 14,000 cases/year in 2018) [1]. Pancreatic cancer was estimated to be the fourth leading cause of cancer deaths in Europe in 2020, with approximately 140,000 new cases and 132,000 deaths [2].

Pancreatic ductal adenocarcinoma (PDAC) constitutes 90% of all pancreatic cancers and could become the second leading cause of cancer-related deaths in Europe between 2030 and 2040 [3].

The prognosis for pancreatic adenocarcinoma remains poor. Fifty percent of cases are detected when the cancer is already at an advanced phase, which at least partially explains the poor survival. The five-year overall survival rate is about 5% in France, increasing to 20% for cases that are truly resectable, i.e., able to be completely removed [1]. Moreover, this poor prognosis is due to PDAC’s resistance to chemotherapy and immunotherapy [4], which is partially attributable to the complex interactions between the tumor and its stromal microenvironment [5]. The discovery of molecular subtypes of pancreatic cancers could help to refine existing therapy and improve patient survival [6]. Bailey et al. identified four molecular subtypes from expression analysis, namely the squamous, pancreatic progenitor, immunogenic and ADEX (aberrantly differentiated endocrine–exocrine) subtypes. The squamous subtype was associated with the worst prognosis [7].

The role of inflammation in cancer growth is well known. Some examples of local inflammation associated with an increased risk of developing cancer are Barrett’s esophagus, inflammatory bowel diseases and chronic pancreatitis [8,9,10]. IL-1α, IL-1β, IL-18, IL-33, IL-36α, IL-36β and IL-36γ are well-known pro-inflammatory cytokines related by the signal transduction pathway utilized (IL-1 common signaling pathway) [11,12]. Local inflammation enhances tumoral progression, invasiveness and immunosuppression by recruiting neutrophils, tumor-associated macrophages, myeloid-derived suppressor cells, regulatory dendritic cells and regulatory T cells [13]. IL-1α and IL-1β promote angiogenesis but also the activation of endothelial cells in a pro-inflammatory direction, creating a microenvironment favorable for cancer promotion. Moreover, they contribute to the suppression of an effective adaptive antitumor immune response by promoting myeloid-derived suppressor cells and sustaining the immunosuppressive function of tumor-associated macrophages [14]. Nevertheless, these cytokines have also shown anti-tumoral activity in some types of cancer [15]. These divergent effects warrant closer attention when developing therapeutic strategies targeting IL-1α and IL-1β.

Using public microarray and RNA sequencing (RNA-seq) datasets, the aim of this study was to investigate the prognostic role of IL-1 and related cytokines in pancreatic cancer patients. More precisely, we evaluated the role of the *IL1A* gene as a prognostic biomarker in pancreatic cancer. Next, the relation between *IL1A* expression and Bailey’s molecular subtypes was investigated. Using single-cell RNA-seq data (scRNA-seq), the expression of *IL1A* was also compared between the different cell populations of the tumor microenvironment, to understand the underlying mechanisms.

## 2. Materials and Methods

### 2.1. Study Design

First, we used scRNA-seq data to confirm the overexpression of certain pro-inflammatory cytokines in pancreatic tumor samples compared to controls.

Then, we sought to evaluate the potential prognostic role of activator markers involved in the IL-1 pathway. We thus performed a retrospective study using two independent cohorts of patients with pancreatic cancer, namely The Cancer Genome Atlas (TCGA, N = 178) [16] cohort and the International Cancer Genome Consortium (ICGC, N = 269) cohort [17]. Samples were selected based on availability of survival and expression data (178 for TCGA cohort, 267 for the ICGC cohort). Survival data included overall survival (OS) for both cohorts, progression-free interval (PFI) for the TCGA cohort and RFS (relapse-free survival) for the ICGC cohort. Analysis was performed using the ICGC cohort as the training set and the TCGA cohort as the validation set.

Next, the relation between *IL1A* expression and Bailey’s molecular subtypes was investigated in both cohorts.

Finally, using scRNA-seq data, we searched for cell populations overexpressing IL-1α in the tumor samples.

### 2.2. Study Population

#### 2.2.1. TCGA Data

RNAseqV2 data (N = 178) with RSEM (RNA-Seq by Expectation-Maximization) normalization and corresponding clinical and survival data were downloaded from the TCGA data portal (https://portal.gdc.cancer.gov/, accessed on 10 May 2022) [16]. For survival analysis, clinical variables such as age, sex, American Joint Committee on Cancer (AJCC) tumoral stage and grading were included.

Survival data, sex and age were available for all 178 patients of the cohort. For AJCC stage and grading, data were available, respectively, for 175 and 176 patients. Bailey’s molecular subtype was available for only 149 individuals.

#### 2.2.2. ICGC Data

Microarray expression data for 269 patients with pancreatic cancer and their survival and clinical data were extracted from the ICGC repository [17]. Expression data were already pretreated and normalized. Survival data were available for 267 patients of the cohort. For survival analysis, clinical variables such as age (N = 266), sex (N = 267), AJCC stage (N = 233) and grading (N = 231) were included. Bailey’s molecular subtype was available for only 88 patients.

#### 2.2.3. Single-Cell RNA-Seq Data

Count data from 24 PDAC patients and 11 control pancreas tissues were obtained from the Genome Sequence Archive [18] under project PRJCA001063 [19].

### 2.3. Single-Cell RNA-Seq Data Analysis

Raw scRNA-seq data were preprocessed using the Seurat R library version 4.0.5 [20].

All Seurat functions were run with default parameters, unless otherwise specified. Low-quality cells with aberrant values of genes/cell (<200 and >7000) and of molecules/cell (>70,000) were excluded, leading us to keep 57,298 cells for further analysis.

Data were then normalized and integrated for downstream analysis. The 2000 most variable genes were identified and used for principal component analysis. The significant principal components previously identified were then used to cluster the cells in different groups using a non-linear dimensional reduction technique (tSNE, t-distributed stochastic neighbor embedding). Labels proposed by Peng et al. [19] were used to phenotype the observed clusters. Finally, the DotPlot function was used to visualize the expression of genes of interest in normal and tumor tissues. The FindMarkers Function (Wilcoxon rank-sum test and Bonferroni correction) was used to compare the expression of *IL1A*, *IL1B*, *IL6*, *TNF*, *IL18* and *IL33* genes between tumor and normal tissue. The same comparison was performed between cell populations in tumor samples for the candidate prognostic marker, i.e., IL1A. 

### 2.4. IL-1 Pathway Analysis

The gene set WP_SIGNAL_TRANSDUCTION_THROUGH_IL1R was used to represent the IL-1 pathway. This gene set was downloaded from the C2 collection of the Molecular Signature database (MSigDB version 7.5) [21,22].

Then, using the expression values of genes composing the IL-1 pathway, we calculated the single-sample Gene Set Enrichment analysis (ssGSEA) [23] scores. The ssGSEA enrichment score represents the activity level of the biological process in which the gene set’s members are coordinately up- or downregulated within a sample.

### 2.5. Statistical Analysis

Continuous variables were compared using the Wilcoxon test. Categorical variables (age, sex, AJCC stage, grade of differentiation) were compared using the chi-squared test. Multi-group comparisons were performed using the Kruskal–Wallis test. 

A *p*-value < 0.05 was considered statistically significant.

The survival R library was used to perform survival analysis. Univariate Cox regression models for OS, PFI and RFS were used to evaluate the prognostic roles of the different variables.

In the TCGA cohort, OS was defined as the time from the date of diagnosis to the date of death from any cause; the censored time was from the date of initial diagnosis to the date of last contact. PFI was defined as the period from the date of diagnosis to the date of the first occurrence of a new tumor event (disease progression, locoregional recurrence, distant metastasis, new primary tumor or death with tumor). Patients who were alive without a new tumor event, or died without a tumor, were censored. The censored time was from the date of diagnosis to the date of last contact or the date of death without a tumor [24].

In the ICGC cohort, OS was defined as the period from the date of diagnosis to the date of death from any cause. If the donor was clinically disease-free after primary treatment and then relapse occurred, RFS was the length of the disease-free interval [25].

Survival probabilities were estimated using the Kaplan–Meier method, and survival curves were compared using the log-rank test. Next, a multivariate Cox proportional hazards regression analysis was performed using the candidate variables identified by the univariate Cox regression analysis. A *p*-value *≥* 0.10 obtained by univariate Cox regression was used as a removal criterion for multivariate Cox regression. Various thresholds were evaluated to identify different groups according to *IL1A* expression. To give a reasonable spread of risk, we distinguished two prognostic groups according to the maximally selected rank statistics, following the Hothorn and Lausen methodology [26]. We chose the first decile cut-off, which is the threshold closest to the maxstat threshold and transposable to the two datasets.

To control the heterogeneity of genomic expression, log2 transformation for the TCGA expression data was adopted. In the ICGC cohort, expression data were already pretreated and normalized; when several probes matched the same gene, the mean of the probe values was used as the expression value of the gene.

Statistical analyses were performed using the R software (version 4.0.3; http://www.R-project.org/, accessed on 29 August 2021), and graphs were drawn using GraphPad Prism version 9.02.

## 3. Results

### 3.1. IL-1-Pathway-Related Gene Expression: Tumor versus Controls

**Figure 1:**
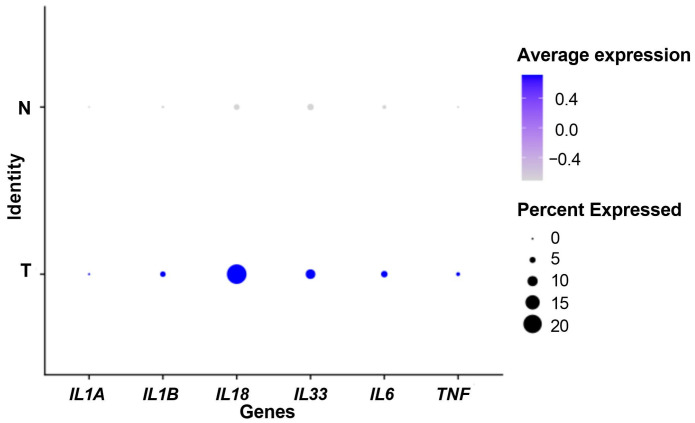
Dot plot showing the expression of IL-1-pathway-related genes. N: control samples. T: tumor samples. Percent expressed: percentage of cells that express the gene. Average expression: average expression using scaled data, negative values correspond to clusters with expression below the mean expression across the whole dataset.

Firstly, we used scRNA-seq data to compare the expression of some genes associated with the IL-1 pathway (*IL1A*, *IL1B*, *IL18*, *IL33*, *IL6*, *TNF*) between 24 PDAC tumoral samples and 11 control pancreas samples. All genes were overexpressed in the tumor samples (Figure 1), suggesting a role for inflammation, particularly for the IL-1 pathway in pancreatic cancer. The Wilcoxon rank-sum tests adjusted using Bonferroni correction were significant for all genes (*p* < 0.001).

### 3.2. Survival Analysis

#### 3.2.1. Patient Characteristics

The clinical characteristics of the patients are shown in Table 1. The training dataset (ICGC) included 267 samples with a median OS of 1.5 years and a median RFS of 1.1 years. The validation dataset was composed of 178 samples (TCGA) with a median OS of 1.7 years and a median PFI of 1.3 years.

No significant differences were found between the two cohorts. However, there was a high rate of missing data for Bailey’s subtypes, especially in the ICGC cohort.

#### 3.2.2. IL-1 Pathway Score as a Prognostic Marker

Univariate Cox regression analysis was performed in both the ICGC and TCGA cohorts to test the prognostic value of age, sex, stage, grade and IL-1 pathway score as continuous variables (Supplementary Tables S1 and S2). The results of the multivariate Cox regression analysis are presented in Table 2 and Table 3.

Regarding overall survival, IL-1 pathway score was an independent prognostic marker of poor survival in the training cohort but not in the validation cohort. As to the risk of recurrence or progression, IL-1 pathway score was an independent prognostic marker in both cohorts.

#### 3.2.3. IL-1α as a Prognostic Marker

Univariate Cox regression analysis was performed to identify cytokines of the IL-1 pathway that were significantly associated with survival, namely we considered genes *IL1A*, *IL1B*, *IL18*, *IL33*, *IL36A*, *IL36B* and *IL36G*. Gene expressions were used as continuous variables (Supplementary Table S3). Only *IL1A* was identified as a prognostic marker in both cohorts for OS (in ICGC: HR= 1.15 [1.07–1.25], *p* < 0.001; in TCGA: HR = 1.10 [1.00–1.21], *p* = 0.044) and for RFS/PFI (in ICGC: HR = 1.13 [1.06–1.21], *p* < 0.001; in TCGA: HR = 1.10 [1.01–1.20], *p* = 0.031). Multivariate Cox models including tumor stage, grading and IL-1α expression were estimated (Supplementary Tables S4 and S5). *IL1A* was found to be an independent prognostic marker for recurrence/progression risk in both cohorts (in ICGC: HR = 1.11 [1.03–1.90] and *p* = 0.009; in TCGA: HR = 1.11 [1.00–1.23], *p* = 0.049). In terms of OS, IL-1α was significantly associated with worse prognosis in the training set only (HR = 1.11 [1.02–1.21], *p* = 0.012), but not in the validation set (HR = 1.09 [0.97–1.22] and *p* = 0.144).

**Figure 2:**
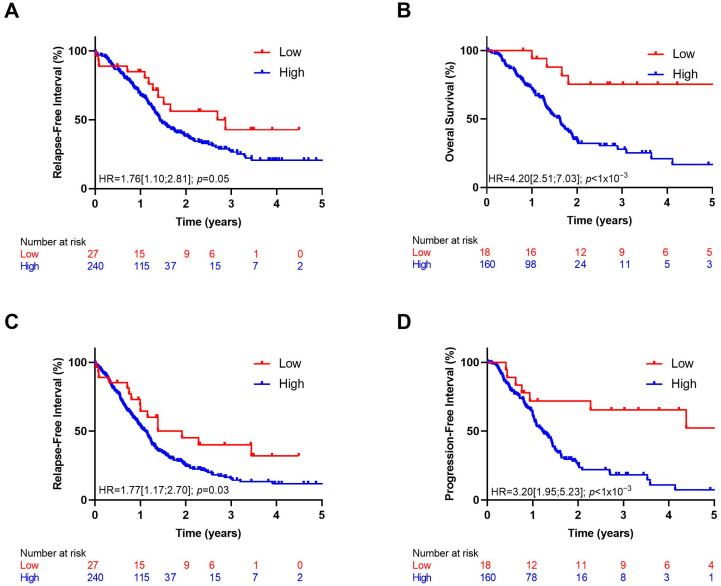
Kaplan–Meier curves with patients stratified according to *IL1A* expression (high: >10% and low: <10%) for overall survival in ICGC (**A**) and TCGA (**B**) cohorts, for relapse-free survival in ICGC cohort (**C**) and progression-free interval in TCGA cohort (**D**).

According to the optimal cut-off percentile value of *IL1A*, Kaplan–Meier curves were estimated in both cohorts. We observed better prognosis among patients in the lowest decile of *IL1A* expression (Figure 2). 

The multivariate Cox analysis results showed that after adjustment for clinical variables such as tumor stage and grade, *IL1A* expression was an independent predictor in both datasets (Table 4 and Table 5).

#### 3.2.4. Bailey’s Molecular Subtypes and IL-1α Expression

**Figure 3:**
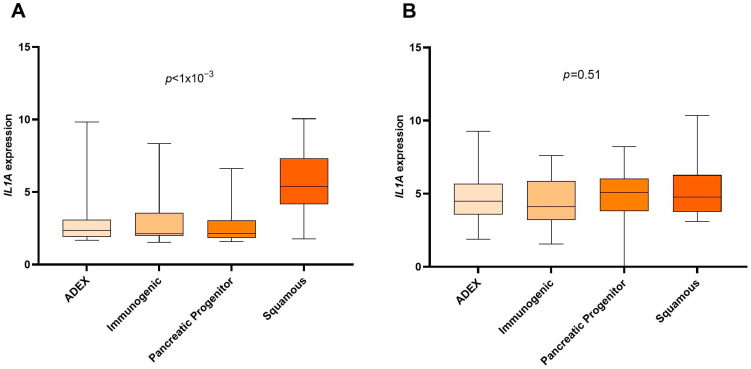
Expression of *IL1A* gene in the different Bailey subtypes. *p*-values were calculated by the Kruskal–Wallis test. (**A**) ICGC cohort. (**B**) TCGA cohort.

To evaluate the prognostic role of Bailey’s subtypes in our datasets, we performed univariate Cox regression between squamous and non-squamous (immunogenic, pancreatic progenitor, ADEX) samples (overall survival). In the ICGC cohort, the squamous subtype was significantly associated with worse prognosis (HR = 2.06 [1.16–3.66], *p* = 0.01), but this was not the case for the validation set (HR = 1.18 [0.71–1.97], *p* = 0.51). In the ICGC cohort, the squamous subtype was also significantly associated with a greater risk of recurrence (HR = 1.80 [1.05–3.08], *p* = 0.03), but in the TCGA cohort, it was not significantly associated with an increased risk of progression/recurrence (HR = 1.56 [0.98–2.47], *p* = 0.06). When comparing the expression of *IL1A* between the different subtypes, we noted that *IL1A* was significantly overexpressed in squamous samples in the training set, but not in the validation set (Figure 3).

### 3.3. IL1A Overexpression in the Tumor Microenvironment

We used scRNA-seq data from 11 normal pancreas and 24 tumor samples to compare the cell composition of both types of samples (Supplementary Figure S1). In normal pancreas samples, we observed a predominance of normal pancreatic cells (ductal cell type 1), while in tumor samples, there was a reduction in normal cells and an increased presence of cancerous cells (ductal cell type 2), with the characteristic accumulation of fibroblasts and immune infiltration.

**Figure 4:**
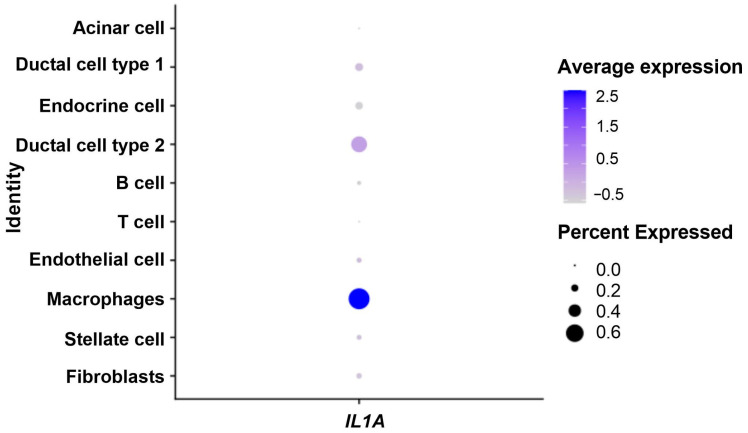
Dot plot showing expression of IL-1α in the tumor microenvironment. Percent expressed: percentage of cells that express *IL1A*. Average expression: average expression using scaled data, where negative values correspond to clusters with expression below the mean expression across the whole dataset.

Then, in tumor samples only, we identified IL-1α production cell populations. As shown in Figure 4, *IL1A* was mostly expressed in macrophages (Wilcoxon test, *p* < 1.10^−3^) and in cancerous cells (Wilcoxon test, *p* = 0.001).

## 4. Discussion

We demonstrate that *IL1A* expression is a prognostic marker in pancreatic cancer, independently of grade and AJCC tumor stage. In fact, all cytokines were more highly expressed in tumors than normal tissue, but among them, only *IL1A* had a prognostic role. The decile of patients with the lowest expression of *IL1A* had better overall survival and a lower risk of recurrence and progression. Increasing *IL1A* expression is significantly associated with a greater risk of recurrence/progression. In fact, activation of the IL-1 signaling pathway is also significantly associated with a higher risk of recurrence/progression.

Previous studies have analyzed the biological role of IL-1α in PDAC. Xu et al. [27] showed that IL-1α expression by metastatic pancreas cell lines induced the production of hepatocyte growth factor (HGF) by fibroblasts, enhancing pancreatic cancer invasion, proliferation and angiogenesis. Another study revealed that the overexpression of IL-1α in non-metastatic pancreatic cancer cell lines constitutively activated nuclear factor-κB (NF-κB) and NF-κB downstream genes involved in the metastatic cascade, promoting metastases [28]. Moreover, the co-culture of cancer-associated fibroblasts with PDAC cells caused the overexpression of IL-1α and other inflammatory genes, helping in the creation of an inflammatory tumor microenvironment conducive to tumor progression. In contrast, the blockade of IL-1α alone resulted in a significant reduction in the expression of other inflammatory genes and could improve the local immune response [29]. All these studies suggest a role of IL-1α in pancreatic cancer progression and a potential impact of its overexpression on cancer outcomes.

Our results also suggest that IL-1α or its receptor Il-1R1 could be a target for therapeutic approaches in PDAC. A previous study underlined that anakinra, an IL-1 receptor antagonist, suppresses tumor growth in mouse models by inhibiting IL-1α-mediated NF-κB activity [30] in pancreatic cancer. Pancreatic stellate cells (PSCs) secrete IL-6, which activates STAT3 and contributes to therapeutic resistance [31,32,33]. Because IL-1α acts upstream of IL-6 release from PSCs, we suspect that IL-1α could promote STAT3 activation within the tumor microenvironment. Furthermore, another study revealed that combining anakinra with chemotherapy significantly improved overall survival in a mouse model of pancreatic ductal adenocarcinoma, thus confirming the deleterious role of IL-1 (PDAC) [34]. Additionally, prior research suggested that blocking the IL-1 pathway with anakinra might also be a promising approach to counteract tumor-induced immunosuppression mediated by C-C-motif ligand 22 (CCL22), a known attractant for regulatory T cells [35].

For our survival analysis, we used survival times available for the ICGC cohort (OS and RFS); however, for TCGA, we used OS and PFI. For TCGA survival analysis, we used PFI and not DFI, because DFI information was only available for 69 out of a total of 178 subjects, causing a substantial loss of statistical power.

Interestingly, our analysis did not confirm that the squamous molecular subtype was a poor prognostic factor [7] in the TCGA cohort. This result could be partially explained by the high rate of missing data for the molecular subtype in our cohorts and the resultant lack of statistical power, even though the missing data were mainly in the ICGC cohort. Furthermore, the squamous subtype was associated with the overexpression of IL1A in the ICGC cohort, but not in the TCGA cohort. These results could suggest a role of IL1A upregulation in worsening the prognosis of squamous samples, but the high number of samples with missing molecular subtype calls for caution in the interpretation of this finding. In a recent study, Somerville et al. [36] showed that enhanced tissue inflammation in pancreatic cancer was a consequence of squamous trans-differentiation. This inflammation is mediated by the transcription factor p63, which activates enhancers at pro-inflammatory cytokine loci, which includes IL1A as a key target. According to this study, overexpression of IL1A in the squamous subtype should be expected. However, in our analysis, this overexpression was found only in the ICGC cohort. Other studies are warranted to investigate IL-1α expression in squamous samples compared to the other molecular subtypes.

Our analysis with single-cell RNA-seq data confirmed the role of inflammation in pancreatic cancer, as IL-1α and other pro-inflammatory cytokines were significantly overexpressed in tumor samples compared to the controls. In the microenvironment of tumor samples, *IL1A* was upregulated in macrophages and cancerous cells. A previous study found that the expression of *IL1A* by tumor cells was detected in 90% of PDAC patients [29]. However, in our analysis, the highest *IL1A* expression was observed in macrophages. A recent meta-analysis revealed that CD68^+^ tumor-associated macrophages (TAMs) in pancreatic cancer were associated with worse overall survival [37]. Thus, the poor prognosis associated with the upregulation of IL-1α could be partially related to tumor infiltration by TAMs. Because *IL1A* is mainly expressed in macrophages, we suspect that the deleterious effect of *IL1A* could also be observed in other tumor types.

Our study has several limitations. This is a retrospective study, and the results would be more robust with a larger sample size. Moreover, other clinical information (for example, the type of treatment) would be useful to better understand the role of IL-1α in patients with pancreatic cancer and explain the differences observed between the two cohorts. In terms of perspectives, experiments in PDAC murine models would also be useful to clarify the role of IL-1α. Several murine pancreatic cancer cell lines, such as Panc02 or KPC can be used to investigate the role of IL-1α in cancer progression. IL-1α can be inhibited with several tools in animal models: IL1a-deficient mice to block the host cytokine, cancer cell lines knocked down for IL-1α expression to inhibit tumor-derived IL-1α, or an anti-IL-1α antibody to target the cytokine, wherever it comes from. Moreover, these tools could be used to investigate whether IL-1α influences the antitumor effect of chemotherapy.

## 5. Conclusions

In this study, we show that *IL1A* gene expression is an independent prognostic marker in pancreatic cancer. The decile of patients with the lowest *IL1A* expression had better overall survival and a lower risk of recurrence and progression. These results highlight the potential of IL-1α as a prognostic marker and a potential therapeutic target in pancreatic cancer.

## Figures and Tables

**Table 1:** Characteristics of the training set and the validation set.

Variables	ICGC Cohort (N = 267)	TCGA Cohort (N = 178)	*p*-Value
Age, N (%)			0.124
<60 years	69 (25.9)	59 (33.1)	
≥60 years	197 (74.1)	119 (66.9)	
Sex, N (%)			0.771
Women	125 (46.8)	80 (44.9)	
Men	142 (53.2)	98 (55.1)	
Tumor stage, N (%)			0.489
IIB + III + IV	176 (75.5)	126 (72)	
IA + IB + IIA	57 (24.5)	49 (28)	
Missing values	34	3	
Grading, N (%)			0.282
Poorly differentiated + undifferentiated	79 (34.2)	50 (28.4)	
Moderately differentiated + well differentiated	152 (65.8)	126 (71.6)	
Missing values	36	2	
Bailey’s subtypes, N (%)			0.077
ADEX	14 (15.9)	38 (25.5)	
Immunogenic	25 (28.4)	27 (18.1)	
Pancreatic progenitor	25 (28.4)	53 (35.6)	
Squamous	24 (27.3)	31 (20.8)	
Missing values	179	29	
Overall survival, median (IQR)	1.5 (2.5)	1.7 (4.6)	
Progression-free interval, median (IQR)	-	1.3 (2.8)	
Relapse-free survival, median (IQR)	1.1 (1.6)	-	

Categorical variables are described as number of observations (%) and were compared between cohorts with the chi-squared test. Survival times are described by median values (years) and interquartile range (IQR). ADEX: aberrantly differentiated endocrine exocrine.

**Table 2:** Multivariate Cox model for overall survival for the ICGC and TCGA cohorts.

Overall Survival				
Variables	ICGC		TCGA	
	HR [95%CI]	*p*-Value	HR [95%CI]	*p*-Value
Stage				
IA + IB + IIA (ref)				
IIB + III + IV	1.47 [0.96–2.25]	0.074	1.85 [1.07–3.20]	**0.028**
Grading				
G1 + G2 (ref)				
G3 + G4	1.63 [1.15–2.30]	**0.006**	1.27 [0.82–1.97]	0.278
IL1 pathway score	4.74 [1.00–22.43]	**0.050**	5.18 [0.85–31.52]	0.074

HR: hazard ratio; CI: confidence interval. (ref) indicates which level was taken as a reference when displaying hazard ratios. *p*-values<0.05 are highlighted in bold.

**Table 3:** Multivariate Cox regression, RFS and PFI.

	Relapse-Free Survival		Progression-Free Interval	
Variables	ICGC		TCGA	
	HR [95%CI]	*p*-Value	HR [95%CI]	*p*-Value
Stage				
IA + IB + IIA (ref)				
IIB + III + IV	1.70 [1.15–2.52]	**0.008**	1.45 [0.90–2.35]	0.124
Grading				
G1 + G2 (ref)				
G3 + G4	1.42 [1.03–1.96]	**0.031**	1.40 [0.92–2.11]	0.112
IL1 pathway score	5.19 [1.27–21.23]	**0.022**	14.88 [2.35–94.21]	**0.004**

HR: hazard ratio; CI: confidence interval. (ref) indicates which level was taken as a reference when displaying hazard ratios. *p*-values < 0.05 are highlighted in bold.

**Table 4:** Multivariate Cox regression for overall survival.

Overall Survival				
Variables	ICGC		TCGA	
	HR [95%CI]	*p*-Value	HR [95%CI]	*p*-Value
Stage				
IA + IB + IIA (ref)				
IIB + III + IV	1.46 [0.95–2.23]	0.082	1.68 [0.97–2.92]	0.065
Grade				
G1 + G2 (ref)				
G3 + G4	1.72 [1.22–2.42]	**0.002**	1.17 [0.75–1.82]	0.482
IL-1A				
<10% (ref)				
≥10%	1.99 [1.01–3.93]	**0.046**	3.00 [1.14–7.90]	**0.026**

HR: hazard ratio; CI: confidence interval. (ref) indicates which level was taken as a reference when displaying hazard ratios. *p*-values < 0.05 are highlighted in bold.

**Table 5:** Multivariate Cox regression for relapse-free survival and progression-free interval.

	Relapse-Free Survival		Progression-Free Interval	
Variables	ICGC		TCGA	
	HR [95%CI]	*p*-Value	HR [95%CI]	*p*-Value
Stage				
IA + IB + IIA (ref)				
IIB + III + IV	1.70 [1.15–2.52]	**0.008**	1.38 [0.85–2.26]	0.196
Grade				
G1 + G2 (ref)				
G3 + G4	1.54 [1.13–2.11]	**0.006**	1.31 [0.86–1.99]	0.212
IL-1A				
<10% (ref)				
≥10%	1.85 [1.02–3.34]	**0.041**	3.11 [1.24–7.80]	**0.015**

HR: hazard ratio; CI: confidence interval. (ref) indicates which level was taken as a reference when displaying hazard ratios. *p*-values < 0.05 are highlighted in bold.

## Data Availability

The original contributions presented in the study are included in the article and Supplementary Material, further inquiries can be directed to the corresponding author.

## Supplementary Materials

The following supporting information can be downloaded at: https://www.mdpi.com/article/10.3390/biomedicines12061216/s1, Table S1: Univariate Cox regression of IL1 pathway score and clinicopathological parameters—OS; Table S2: Univariate Cox regression of IL1 pathway score and clinicopathological parameters—RFS, PFI; Table S3: Univariate Cox regression of IL-1 common signaling pathway activators—OS, RFS and PFI; Table S4: Multivariate Cox regression—OS; Table S5: Multivariate Cox regression—RFS, PFI; Figure S1: Cell populations in normal (N) and tumor (T) samples.
