# Supplementary material for: Interleukin-1α as a Potential Prognostic Biomarker in Pancreatic Cancer
Source: Biomedicines. 2024 May 30;12(6):1216. doi: 10.3390/biomedicines12061216 (PMC11200603; doi:10.3390/biomedicines12061216)
Supplement: Supplementary file 1 [file biomedicines-12-01216-s001.zip › biomedicines-2979519-supplementary.pdf]

Table S1. Univariate Cox regression of IL1 pathway score and clinicopathological parameters - OS

	**Overall Survival**					
**Variables**	**ICGC**			**TCGA**		
	**N**	**HR [95%CI]**	***p*-value**	**N**	**HR [95%CI]**	***P-value***
**Age at diagnosis**	266			178	1.42 [0.91-2.23]	0.123
≤60 (ref)	69			59		
>60	197	0.92 [0.64-1.31]	0.643	119		
**Sex**	267					
Male (ref)	142			98		
Female	125	0.81 [0.60-1.12]	0.204	178	1.21 [0.81-1.82]	0.350
**Stage**	233					
IA+IB+IIA (ref)	57			49		
IIB+III+IV	176	1.44 [0.95-2.16]	0.082	175	2.09 [1.24-3.52]	**0.005**
**Grade**	230					
G1+G2 (ref)	152			126		
G3+G4	78	1.72 [1.22-2.42]	**0.002**	176	1.52 [0.98-2.34]	0.060
**IL1 pathway score**	267	5.47 [1.27-23.53]	**0.022**	178	13.76 [2.58-73.27]	**0.002**

HR: hazard ratio; CI: confidence interval. The (ref) indicates which level was taken as a reference when displaying hazard ratios. P-values<0.05 are highlighted in bold

Table S2. Univariate Cox regression of IL1 pathway score and clinicopathological parameters – RFS, PFI

	**Relapse Free Survival**			**Progression Free Interval**		
**Variables**	**ICGC**			**TCGA**		
	**N**	**HR [95%CI]**	***p*-value**	**N**	**HR [95%CI]**	***p*-value**
**Age at diagnosis**	266 (1 NA)					
≤60 (ref)	69			59		
>60	197	0.90 [0.65-1.24]	0.514	178	0.94 [0.64-1.40]	0.781
**Sex**	267					
Male (ref)	142			98		
Female	125	0.89 [0.68-1.18]	0.403	178	1.03 [0.70-1.51]	0.894
**Stage**	233 (34 NA)					
IA_IB_IIA (ref)	57			49		
IIB_III_IV	176	1.69 [1.15-2.47]	**0.007**	175 (3 NA)	1.93 [1.21-3.10]	**0.006**
**Grade**	230 (37 NA)					
G1_G2 (ref)	152			126		
G3_G4	78	1.56 [1.14-2.13]	**0.005**	176 (2 NA)	1.67 [1.11-2.53]	**0.015**
**IL1 pathway score**	267	6.34 [1.72-23.31]	**0.005**	178	22.19 [4.15-118.74]	**< 0.001**

HR: hazard ratio; CI: confidence interval. The (ref) indicates which level was taken as a reference when displaying hazard ratios. P-values<0.05 are highlighted in bold

Table S3. Univariate Cox regression of IL-1 common signaling pathway activators - OS, RFS and PFI. Gene expressions are used as continuous variable.

	Univariate Cox Regression, Continuous Variables							
	Overall Survival				Relapse Free Survival		Progression Free Interval	
**Variable**	**ICGC**		**TCGA**		**ICGC**		**TCGA**	
	**HR [95%CI]**	**P-value**	**HR [95%CI]**	***P-value***	**HR [95%CI]**	***P-value***	**HR [95%CI]**	***P-value***
IL1A	1.15 [1.07-1.25]	**< 0.001**	1.10 [1.00-1.21]	**0.044**	1.13 [1.06-1.21]	**< 0.001**	1.10 [1.01-1.20]	**0.031**
IL1B	0.98 [0.90-1.08]	0.745	1.03 [0.90-1.17]	0.706	0.98 [0.90-1.07]	0.666	0.99 [0.87-1.12]	0.893
IL18	1.05 [0.81-1.36]	0.713	1.59 [1.25-2.02]	**< 0.001**	1.13 [0.89-1.44]	0.316	1.58 [1.27-1.96]	**< 0.001**
IL33	0.72 [0.59-0.88]	**0.002**	0.97 [0.85-1.12]	0.706	0.77 [0.64-0.93]	**0.007**	0.97 [0.84-1.11]	0.636
IL36A	0.99 [0.64-1.54]	0.98	1.20 [0.47-3.08]	0.71	0.94 [0.63-1.39]	0.76	1.07 [0.45-2.58]	0.87
IL36B	1.63 [1.03-2.57]	**0.04**	1.11 [0.93-1.32]	0.23	1.45 [0.95-2.22]	0.09	1.18 [1.01-1.37]	**0.03**
IL36G	1.02 [0.81-1.30]	0.85	1.04 [0.89-1.22]	0.63	1.08 [0.88-1.33]	0.44	0.95 [0.81-1.12]	0.57

HR: hazard ratio; CI: confidence interval. The (ref) indicates which level was taken as a reference when displaying hazard ratios. P-values<0.05 are highlighted in bold

Table S4. Multivariate Cox regression - OS. IL-1α expression is used as continuous variable.

	Overall Survival			
**Variables**	**ICGC**		**TCGA**	
	**HR [95%CI]**	***P-value***	**HR [95%CI]**	***P-value***
**Stage**				
IA+IB+IIA (ref)				
IIB+III+IV	1.57 [1.03-2.40]	**0.037**	2.02 [1.18-3.45]	**0.010**
**Grade**				
G1+G2 (ref)				
G3+G4	1.52 [1.06-2.18]	**0.022**	1.26 [0.81-1.95]	0.305
**IL1A**	1.11 [1.02-1.21]	**0.012**	1.09 [0.97-1.22]	0.144

HR: hazard ratio; CI: confidence interval. The (ref) indicates which level was taken as a reference when displaying hazard ratios. P-values<0.05 are highlighted in bold

Table S5. Multivariate Cox regression – RFS, PFI. IL-1α expression is used as continuous variable.

	Relapse Free Survival		Progression Free Interval	
**Variables**	**ICGC**		**TCGA**	
	**HR [95%CI]**	***P-value***	***P-value***	***P-value***
**Stage**				
IA+IB+IIA (ref)				
IIB+III+IV	1.85 [1.24-2.74]	**0.002**	1.67 [1.04-2.68]	**0.034**
**Grade**				
G1+G2 (ref)				
G3+G4	1.35 [0.97-1.89]	0.077	1.39 [0.91-2.10]	0.125
**IL1A**	1.11 [1.03-1.90]	**0.009**	1.11 [1.00-1.23]	**0.049**

HR: hazard ratio; CI: confidence interval. The (ref) indicates which level was taken as a reference when displaying hazard ratios. P-values<0.05 are highlighted in bold

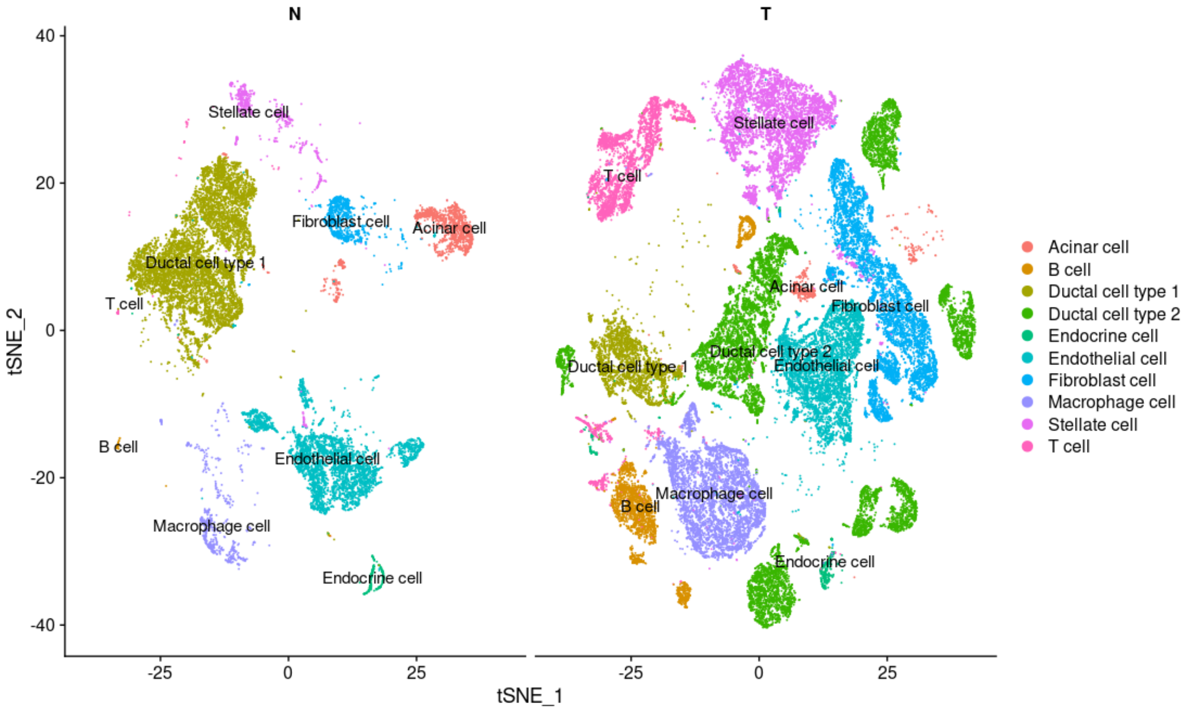

Figure S1. Cell populations in normal (N) and tumor (T) samples.
